# A Nonlinear Relationship Between ALT Levels at Delivery and the Risk of Postpartum ALT Flares in Pregnant Women with Chronic Hepatitis B

**DOI:** 10.7150/ijms.79663

**Published:** 2023-01-22

**Authors:** Mingfang Zhou, Haodong Cai, Wei Yi, Xuesong Gao

**Affiliations:** 1 Department of Obstetrics and Gynecology, Beijing Ditan Hospital, Capital Medical University, Beijing, 100015, China.; 2 Hepatology clinic, Beijing Ditan Hospital, Capital Medical University, Beijing, 100015, China.; 3 Department of General Medicine, Beijing Ditan Hospital, Capital Medical University, Beijing, 100015, China.

**Keywords:** Hepatitis B virus, Alanine Transaminase, Postpartum Period, Retrospective Studies, Pregnancy, Liver Function Tests

## Abstract

**Background:** The aim of the present study was to investigate the association between alanine aminotransferase (ALT) levels at delivery and postpartum ALT flares among women with chronic hepatitis B (CHB).

**Methods:** Pregnant women with CHB from November 2008 to November 2017 were included in this retrospective study. Multivariable logistic regression analysis and a generalized additive model were performed to determine both linear and nonlinear relationships between ALT levels at delivery and postpartum ALT flares. Stratification analysis was performed to test for effect modifications in subgroups.

**Results:** A total of 2643 women were enrolled. Multivariable analysis indicated that ALT levels at delivery were positively associated with postpartum ALT flares (odds ratio (OR) 1.02, 95% confidence interval (CI) 1.01-1.02, P < 0.0001). When ALT levels were converted to a categorical variable, the ORs and 95% CIs in quartiles 3 and 4 versus quartile 1 were 2.26 (1.43-3.58) and 5.34 (3.48-8.22), respectively (P for trend < 0.001). When ALT levels were dichotomized into a categorical variable according to clinical cutoffs (40 U/L or 19 U/L), the ORs and 95% CIs were 3.06 (2.05-4.57) and 3.31 (2.53-4.35), respectively (P < 0.0001). The ALT level at delivery was also found to have a nonlinear relationship with postpartum ALT flares. The relationship followed an inverted U-shaped curve.

**Conclusions:** The ALT level at delivery was positively correlated with postpartum ALT flares in women with CHB when the ALT level was less than 182.8 U/L. The ALT cutoff (19 U/L) at delivery was more sensitive to predict the risk of ALT flares postpartum.

## Introduction

Chronic hepatitis B (CHB) is a serious public health problem worldwide. Overall, approximately 257 million people live with HBV infection, including 65 million women of childbearing age. Mother-to-child transmission (MTCT) is the major route of hepatitis B virus (HBV) transmission 1. Nucleos(t)ide analog (NA) therapy is recommended for mothers with high HBV DNA loads during the second or third trimester 2,3.

Pregnancy and the postpartum period are associated with unique changes in the immune system. During pregnancy, cell-mediated immunity is suppressed, allowing the woman's body to tolerate the fetus. The immunosuppressive effect is reversed after delivery 4. These changes in immunity have long been known to be associated with clinical consequences. Alanine aminotransferase (ALT) flares during pregnancy or after delivery are more common in HBV-infected women 5-9. Although severe cases, including liver failure and even liver transplantation, have been reported during pregnancy, most postpartum ALT flares were mild to moderate and resolved spontaneously 6,10,11. The reported frequency of postpartum ALT flares was 0-36% in untreated mothers and 5.6%-61.5% in treated mothers, including mothers with NA cessation 12,13. The results were highly variable because of different definitions of ALT flares, patients' demographic characteristics, whether antiviral therapy (AVT) was received during pregnancy, the duration of AVT, and limited sample sizes.

It is important to identify risk factors for improvement in the clinical outcomes of women with postpartum ALT flares. However, predictors for postpartum ALT flares remain controversial. Elevated ALT levels at delivery 14 or at 32 weeks of gestation 15 were positively associated with an increased risk of postpartum ALT flares in women with CHB, but none of the risk factors have been demonstrated in other studies 7,10,16,17. Furthermore, there are no definite optimal ALT thresholds or reference intervals predicting postpartum ALT flares. Therefore, we investigated the association of ALT levels at delivery with the risk of postpartum ALT flares in a large-scale retrospective study of women with CHB.

## Patients and methods

### Study design and population

In this retrospective cohort study, pregnant women with CHB who visited hepatology clinics or obstetrics clinics in Beijing Ditan Hospital, Capital Medical University from November 2008 to November 2017 were included. Patients were diagnosed with CHB according to the Guideline of Prevention and Treatment for Chronic Hepatitis B (2015 Update) 3.

All pregnant women were screened from the database we built previously 9, which enrolled HBsAg-positive persons of childbearing age and excluded the following: (1) pregnant women with major systemic disease, including renal insufficiency, heart disease, and malignant neoplasm; (2) those with coinfection with hepatitis C virus, hepatitis D virus, human immunodeficiency virus, toxoplasmosis, syphilis, rubella, or cytomegalovirus; (3) those with other causes of hepatitis, including alcohol abuse, drug-induced liver injury, acute fatty liver of pregnancy and cholestasis of pregnancy; and (4) those with incomplete data at delivery or postpartum.

All pregnant women were enrolled before delivery. All mothers were followed up at 6, 12 and 28 weeks postpartum. The eligibility criteria were as follows: (1) HBsAg-positive mothers; (2) mothers with at least two data points for liver function tests of 6, 12, and 24 weeks postpartum; and (3) mothers who underwent at least one liver function test after AVT withdrawal if they stopped AVT after delivery. For women who had multiple deliveries, each delivery was included as a separate observation.

### Data collection

The following baseline demographic and clinical data were collected from the electronic medical record system of Beijing Ditan Hospital: age, parity, whether AVT was received before pregnancy, ALT levels, aspartate aminotransferase (AST) levels, HBeAg status, HBV DNA viral load, whether AVT was received during pregnancy, the mode of delivery, and treatment status after delivery. A postpartum ALT flare was defined as a serum ALT level ≥ 5 times the upper limit of normal (ULN=40 U/L) within 28 weeks postpartum. For women who had postpartum ALT flares that had not resolved by the second follow-up test, only one ALT flare was counted.

### Statistical analysis

Categorical variables are presented as numbers and percentages and were compared by chi-square analysis (Fisher's exact test if needed). Normally distributed continuous variables are expressed as the mean ± standard deviation (SD), whereas those with nonnormal distributions are expressed as medians and interquartile ranges (IQRs). To evaluate the risk of postpartum ALT flares according to different levels of ALT, we divided patients into quartiles based on ALT distribution. Logistic regression was used to calculate the risk ratio (OR) and 95% confidence interval (95% CI) of postpartum ALT flares in patients. Multivariable logistic models were used to evaluate the associations between ALT levels at delivery and postpartum ALT flares. The crude model was not adjusted for any variables. Model I was adjusted for age; Model II was adjusted for age, parity, whether antiviral therapy was received before pregnancy, HBeAg status, whether antiviral therapy was received during pregnancy, HBV DNA viral load, cesarean section, and the maintenance of AVT, cessation of AVT or no AVT after delivery. The association between ALT levels at delivery and postpartum ALT flares was evaluated using the ALT level as both a continuous variable and a categorical variable. Generalized additive models (GAMs) were used to identify nonlinear relationships between ALT levels at delivery and postpartum ALT flares. A two-piecewise linear regression model was used to calculate the threshold effect of ALT levels at delivery and postpartum ALT flares according to the smoothing plot. The threshold value of ALT levels at delivery, at which the association between ALT levels at delivery and postpartum ALT flares began to change and became noticeable, was determined by applying a trial method. With this method, the trial inflection point is moved along a predefined interval to detect a distinct inflection point that provides the maximum model likelihood 18. We also conducted a log likelihood ratio test comparing the one-line linear regression model with a two-piecewise linear model. Subgroup analyses were performed using stratified logistic regression models. A two-tailed P < 0.05 was considered statistically significant.

Data were analyzed with the use of the statistical packages R (The R Foundation; http://www.r-project.org; version 3.4.3) and EmpowerStats (www.empowerstats.com, X&Y solutions, Inc. Boston, Massachusetts).

## Results

### Baseline characteristics of the patients

**Figure 1:**
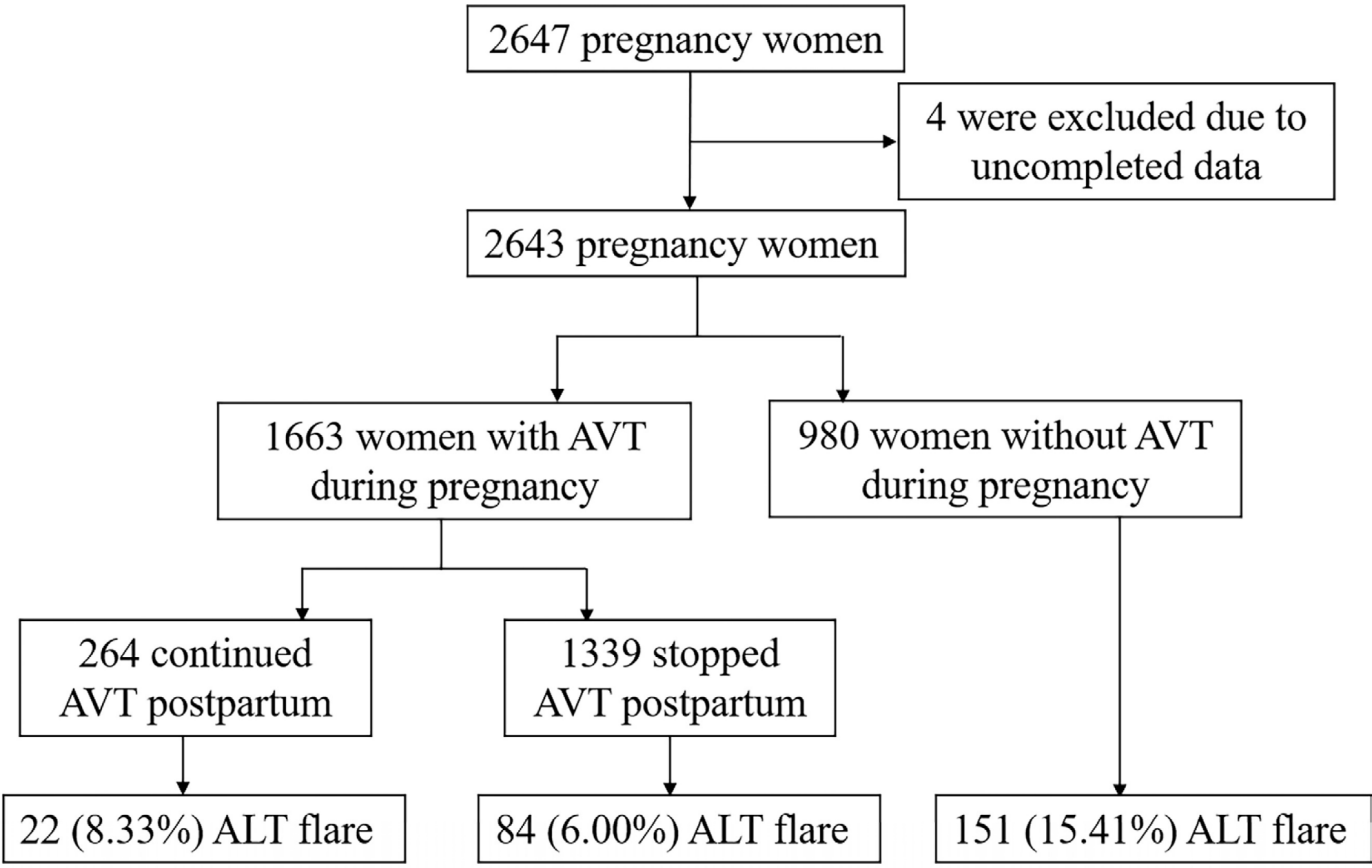
Flowchart of the enrollment of pregnant women with HBV.

A total of 2647 HBsAg-positive pregnant women were screened. Four women were excluded due to incomplete data (Figure 1). Data were divided into quartiles according to ALT levels at delivery (U/L) [Q1 (<11.7), Q2 (11.7-15.5), Q3 (15.5-21.8) and Q4 (≥21.8)]. Their baseline characteristics are shown in Table 1. Overall, the age of the patients was 28.0 ± 4.0 years. The median baseline ALT level was 15.5 U/L (11.7-21.8 U/L). The median HBV DNA level was 3.89 (2.73-6.35) Log_10_ IU/mL. Nearly all women (97.84%) were HBeAg-positive. One thousand six hundred and sixty-three women (62.92%) received AVT during pregnancy, including 364 women who were treated with lamivudine, 1284 with telbivudine, and 15 with tenofovir disoproxil fumarate. One hundred seventy (6.43%) women had abnormal ALT levels (>40 IU/L). Significant differences were observed across all quartiles of ALT levels for age, the proportion of HBeAg positivity, and AVT administration before pregnancy (P <0.001). Patients with the highest ALT level in the top quartile (Q4) were more likely to be older and receive AVT before pregnancy (P <0.001). No significant differences were detected for HBV DNA viral load, parity, AVT administration during pregnancy, or cesarean section across the ALT quartiles.

### Postpartum ALT flares and predictors of ALT flares

**Figure 2:**
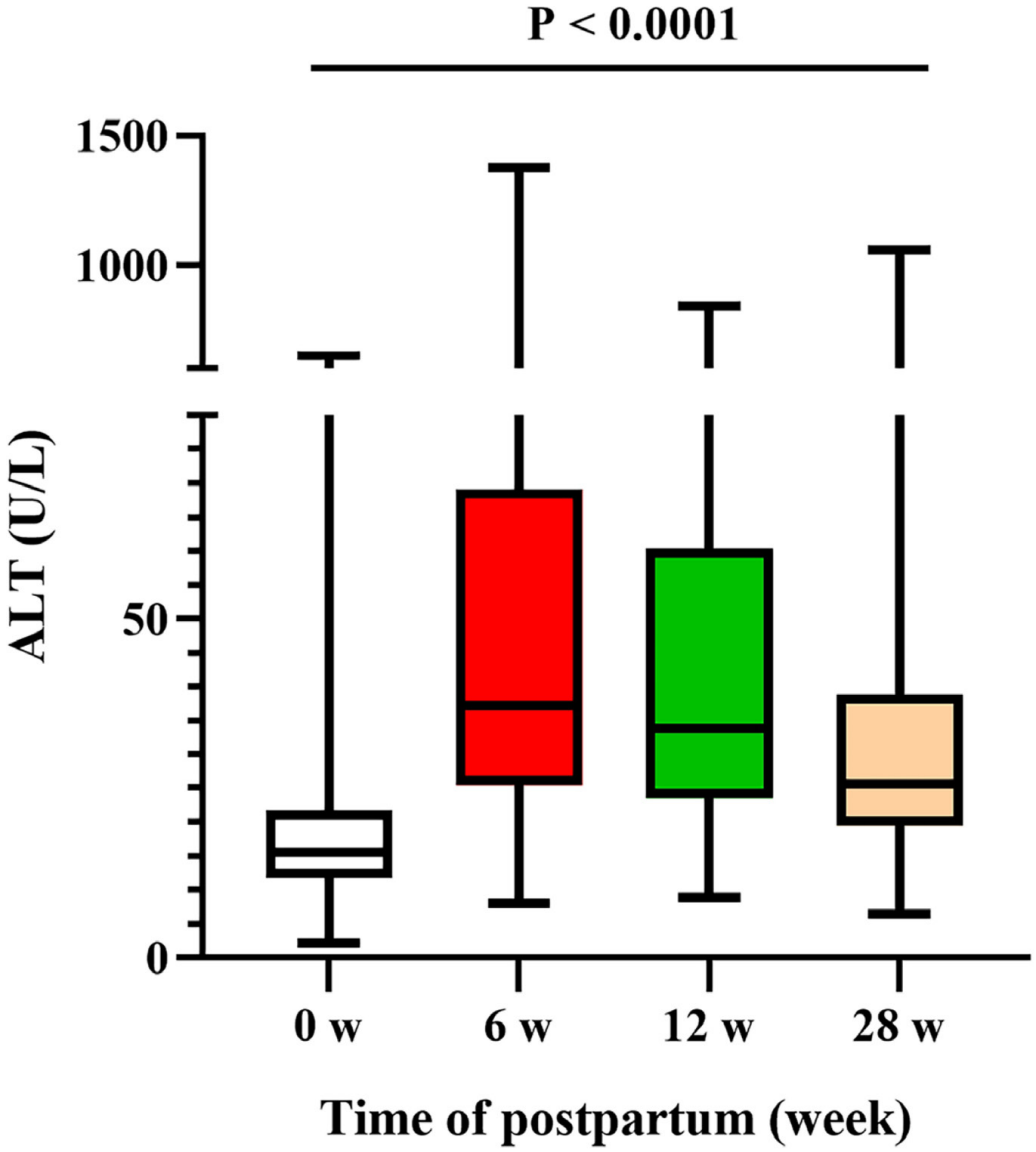
ALT levels during the postpartum period.

ALT flares occurred in 9.72% (257/2643) of the patients during the postpartum period: 170/2613 patients had flares at 6 weeks postpartum (range 200.3-1378.5), 98/2318 had flares at 12 weeks postpartum (range 201.5-842.3), and 43/1889 had flares at 28 weeks postpartum (range 202.2-1060.6). Among the patients who received AVT during pregnancy, 8.33% (22/264) who continued to receive AVT after delivery developed ALT flares, and 6.00% (84/1399) who stopped AVT after delivery developed ALT flares. Seventy-three women occurred ALT flares after the discontinuation of AVT. Comparatively, 15.41% (151/980) of the patients who did not receive AVT during pregnancy developed ALT flares. ALT trends from the time of delivery (0 weeks) to 28 weeks postpartum are shown in Figure 2 (P <0.0001).

### Multivariable logistic regression analysis of ALT levels at delivery and postpartum ALT flares

In multivariable regression analysis, the ALT level at delivery (OR 1.02, 95% CI 1.01-1.02, P < 0.0001) was associated with the risk of postpartum ALT flares. ALT levels were treated as a categorical variable (quartiles and clinical cutoffs) for sensitivity analysis. In the three distinct models, taking Q1 as a reference, women in Q3 (15.5-21.8 U/L) and Q4 (>21.8 U/L) had a substantially higher risk of postpartum ALT flares. After adjusting for age, parity, AVT administration before pregnancy, HBeAg status, AVT administration during pregnancy, HBV DNA viral load, cesarean section, and postpartum treatment status, women in Q3 and Q4 still had a 2.26-fold (OR 2.26, 95% CI 1.43-3.58) and 5.34-fold (OR 5.34, 95% CI 3.48-8.22) higher risk of developing ALT flares postpartum, respectively (all P for trend < 0.05). The nonequally spaced change in the categorical variable of ALT levels indicated the possibility of a nonlinear relationship between ALT levels at delivery and postpartum ALT flares. When ALT levels were dichotomized into a categorical variable according to clinical cutoffs (40 U/L or 19 U/L), the ORs and 95% CIs were 3.06 (2.05-4.57) and 3.31 (2.53-4.35), respectively (P < 0.0001) (Table 2).

### Threshold Effect Analysis of ALT levels and postpartum ALT flares

**Figure 3:**
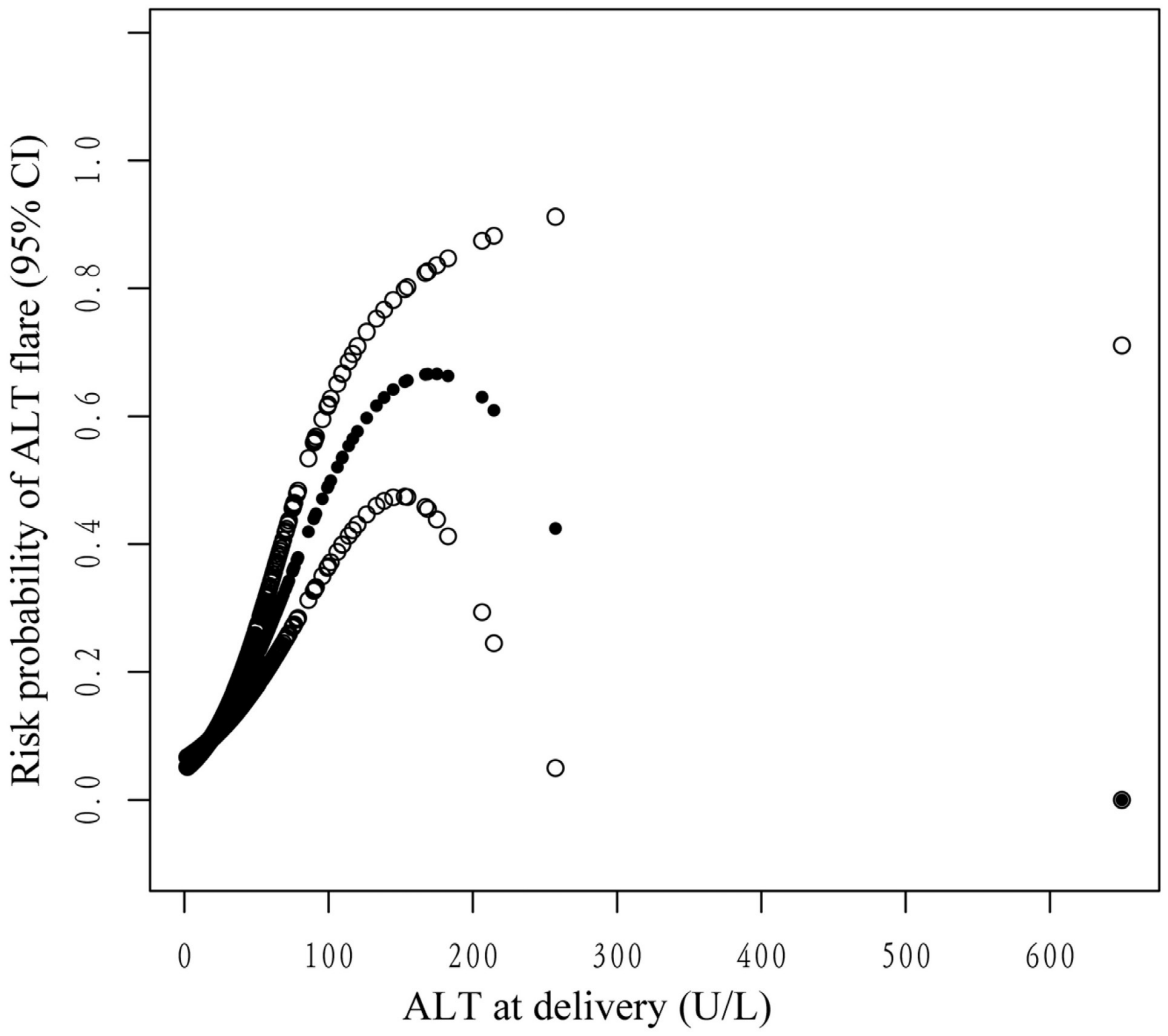
The nonlinear relationship between ALT levels at delivery and the probability of postpartum ALT flares. Adjusted for age, parity, whether antiviral therapy was received before pregnancy, HBeAg status, whether antiviral therapy was received during pregnancy, HBV DNA viral load, cesarean section, and treatment status postpartum. The y-axis represents the probability of postpartum ALT flares. The x-axis represents ALT at delivery. The solid and soft dots represent the estimated value and its corresponding 95% confidence interval, respectively.

Smooth curve fitting and generalized additive models were utilized to explore the potential nonlinear connection between ALT levels at delivery and ALT flares postpartum. As shown in the smoothing spline, ALT levels were indicated to have a nonlinear relationship with the incidence of postpartum ALT flares. It was an inverted U-shape curve (Figure 3). The two-piecewise regression model showed that the inflection point was 182.8 U/L after fully adjusting for covariates. On the left side of the inflection point (ALT < 182.8 U/L), the incidence of postpartum ALT flares increased by 2% per 1 U/L increase in the ALT level (OR 1.02, 95% CI 1.02-1.03, P <0.001) (Table 3). On the right side of the inflection point (ALT > 182.8 U/L), the effect size had no statistical significance (OR 0.99; 95% CI 0.97-1.01; P = 0.2151). However, there were only four mothers with ALT > 182.8 U/L at delivery.

### Subgroup analyses by covariates

To further understand other possible influencing factors in the risk of postpartum ALT flares, we performed stratified analyses by subgroups defined by all covariates (Table 4). Statistically significant interactions were not found for age, parity, whether AVT was received before pregnancy, whether AVT was received during pregnancy, HBV DNA viral load, cesarean section, or postpartum treatment status. We observed that the risk of ALT flares was increased in women in subgroups with ORs ranging from 1.00 to 1.04, except for women who were HBeAg-negative (OR 0.99, 95% CI, 0.95-1.03). The prediction of ALT flares was not statistically significant among HBeAg-negative patients (P = 0.6197).

## Discussion

We found that ALT flares, defined as ALT levels ≥ 5 times the ULN, developed in 9.72% of the HBV-infected women after delivery. Our study demonstrated a significant association between ALT levels at delivery and ALT flares within 28 weeks postpartum. Moreover, two piecewise linear regression analyses were performed, and the results showed a nonlinear relationship between ALT levels at delivery and the risk of postpartum ALT flares.

To date, the independent predictors of postpartum ALT flares remain elusive. Yi et al. found that elevated ALT levels (>40 U/L) at delivery were an independent risk factor for postpartum ALT flares, using similar ALT flare thresholds 14. Quan et al. found that the ALT level at 32 weeks of gestation was an independent predictor (OR 1.067, 95% CI 1.036-1.0993, P <0.001) of postpartum ALT flares. However, postpartum ALT flares were defined as ALT levels greater than 40 U/L within 6 weeks postpartum in their study 15. Liu et al. found that elevated ALT levels (>40 U/L) during pregnancy correlated with postpartum flares after telbivudine withdrawal at 12 weeks postpartum (OR 7.553, 95% CI 1.236-46.153, P=0.029) 19. However, their research lacked further analysis of the relationship between ALT levels at delivery and ALT flares postpartum. We also chose women's data at delivery as the baseline characteristics and demonstrated that the ALT level at delivery was an independent risk factor for ALT flares postpartum. When we treated ALT levels as a categorical variable according to the different clinical cutoffs of 40 U/L or 19 U/L, the risk of developing ALT flares after delivery was similar. Therefore, the ALT cutoff (19 U/L) was more likely to predict the risk of ALT flares earlier. Furthermore, their relationship was nonlinear. On the left side of the inflection point (ALT levels <182.8 U/L), the incidence of postpartum ALT flares increased by 2% per 1 U/L increase in the ALT level (P < 0.0001). When the ALT level exceeded 182.8 U/L, the effect size was not statistically significant. We speculated that if ALT levels were too high at delivery, the patients might be given treatment to protect against liver damage. Therefore, the risk of ALT flares after delivery was reduced instead. However, the number of mothers with ALT > 182.8 U/L at delivery was too small in our cohort, which might affect the statistical analysis. Further studies with a larger sample size are needed to determine the impact of higher ALT levels at delivery on the risk of ALT flares postpartum.

ALT flares were observed in 10% (5/51) of the women within the first 3 months postpartum in the Chang et al. report 10. Gile found that 25% of women (27/108) developed a postpartum flare 17. However, no independent predictors were found in their studies, including age, HBeAg positivity, HBV DNA viral load, ALT, gravidity, and parity. Biochemical or virological characteristics at delivery were not included in either of the aforementioned studies. Bzowej found that 17.2% (5/29) of women developed ALT flares within 14 weeks of drug discontinuation 20. Postpartum ALT flares were observed in 33% (3/9) of women who stopped treatment at delivery in Chang's other study. They considered that ALT flares were more common among patients who discontinued AVT postpartum 16. However, they also failed to demonstrate any independent predictors associated with postpartum ALT flares. Our diverse findings might be due to differences in our patients, including a large sample size, a large number of patients receiving AVT during pregnancy, a majority of HBeAg-positive patients, or the duration of AVT before discontinuation, and the different baseline definitions, which might reflect different activity in the context of pregnancy.

Our study had several strengths. First, to the best of our knowledge, this is the first observation of a nonlinear relationship between ALT levels at delivery and the risk of postpartum ALT flares in mothers with CHB. Second, we developed the rationale behind the nonlinearity of ALT levels at delivery and the risk of postpartum ALT flares in the present study. Third, the results of this study should be useful in carrying out intensive surveillance for women with different ALT levels to determine their risk of postpartum ALT flares, particularly those with ALT levels > the ULN. Compared with the ALT cutoff 40 U/L, a cut-off value of 19 U/L was more likely to predict the risk of ALT flares earlier.

There are some limitations to this study. First, as postpartum obstetric visits were scheduled at 6, 12 weeks and 28 weeks after delivery, we could not calculate the cumulative incidence of ALT flares at different weeks after delivery. Second, there was the possibility that ALT flares without symptoms occurring between two outpatient visits would have been missed. Third, there is no general accepted definition of a postpartum ALT flare; therefore, we used a fivefold increase in ALT levels as the definition. Finally, most of our research participants were women with HBeAg positive. It is uncertain whether this conclusion can be extended to all pregnant women.

## Conclusion

ALT levels at delivery were significantly and independently predictive of ALT flares within 28 weeks postpartum in women with CHB. The ALT cutoff (19 U/L) at delivery was more sensitive to predict the risk of ALT flares postpartum. The relationship between ALT levels at delivery and the risk of postpartum ALT flares was nonlinear. An ALT level < 182.8 U/L at delivery was positively associated with the risk of ALT flares postpartum.

## Figures and Tables

**Table 1:** Baseline characteristics of the patients in the cohort

Characteristics	Overall	ALT levels (U/L)				P value
		Q1 (<11.7)	Q2 (11.7-15.5)	Q3 (15.5-21.8)	Q4 (≥21.8)	
N	2643	655	660	663	665	
Age	28.0 ± 4.0	27.56 ± 4.08	27.61 (3.84)	28.21 (4.04)	28.53 (3.99)	<0.001
ALT (U/L)	15.5 (11.7-21.8)	9.8 (8.5-10.7)	13.5 (12.6-14.4)	17.9 (16.4-19.6)	29.8 (25.0-40.4)	<0.001
AST (U/L)	19.9 (16.4-25.5)	15.8 (14.1-17.9)	18.3 (16.1-20.9)	21.1 (18.7-24.6)	31.8 (25.4-40.4)	<0.001
HBVDNA (Log_10_ IU/mL)	3.89 (2.73-6.35)	3.87 (2.65-6.26)	3.95 (2.81-6.53)	4.02 (2.80-6.54)	3.75 (2.68-6.01)	0.125
Parity (≥1)	557 (21.07%)	133 (20.31%)	140 (21.21%)	133 (20.06%)	151 (22.71%)	0.633
HBeAg (+)	2586 (97.84%)	643 (98.17%)	654 (99.09%)	651 (98.19%)	638 (95.94%)	<0.001
AVT before pregnancy	81 (3.06%)	10 (1.53%)	14 (2.12%)	18 (2.71%)	39 (5.86%)	<0.001
AVT during pregnancy	1663 (62.92%)	410 (62.60%)	406 (61.52%)	405 (61.09%)	442 (66.47%)	0.163
Abnormal ALT at delivery (>40 U/L)	170 (6.43%)	0 (0.00%)	0 (0.00%)	0 (0.00%)	170 (25.56%)	<0.001
Cesarean section	1129 (42.72%)	289 (44.12%)	282 (42.73%)	282 (42.53%)	276 (41.50%)	0.816

Note: ALT alanine aminotransferase, AST aspartate aminotransferase, AVT antiviral therapy.

**Table 2:** Relationship between ALT levels at delivery and ALT flares postpartum in different models

	Crude model OR (95% CI)	P Value	Model, I OR (95% CI)	P Value	Model II, OR (95% CI)	P Value
ALT at delivery (U/L)	1.02 (1.01, 1.02)	<0.0001	1.02 (1.01, 1.02)	<0.0001	1.02 (1.01, 1.02)	<0.0001
**ALT at delivery (quartiles)**						
Q1	Reference		Reference		Reference	
Q2	1.32 (0.80, 2.17)	0.2740	1.32 (0.80, 2.16)	0.2744	1.31 (0.79, 2.16)	0.2920
Q3	2.31 (1.47, 3.63)	0.0003	2.30 (1.46, 3.62)	0.0003	2.26 (1.43, 3.58)	0.0005
Q4	5.05 (3.32, 7.68)	<0.0001	5.02 (3.30, 7.65)	<0.0001	5.34 (3.48, 8.22)	<0.0001
P for trend	<0.0001		<0.0001			<0.0001
**ALT clinical cutoffs**						
≤ 40 U/L	Reference		Reference		Reference	
> 40 U/L	3.20 (2.19, 4.68)	<0.0001	3.16 (2.16, 4.63)	<0.0001	3.06 (2.05, 4.57)	<0.0001
≤ 19	Reference		Reference		Reference	
>19	3.27 (2.51, 4.26)	<0.0001	3.26 (2.50, 4.25)	<0.0001	3.31 (2.53, 4.35)	<0.0001

Note: Crude model: did not adjust covariates. Model I: adjusted for age. Model Ⅱ: adjusted for age, parity, AVT before pregnancy, HBeAg, AVT during pregnancy, HBVDNA viral load, cesarean section, treatment status postpartum.

**Table 3:** Two-piecewise linear regression analysis for ALT levels at delivery and postpartum ALT flares

The Inflection Point of ALT (U/L)	OR (95% CI)	P value
< 182.8	1.02 (1.02, 1.03)	<0.0001
> 182.8	0.99 (0.97, 1.01)	0.2151
Log likelihood ratio test		<0.001

Note: Adjusted for age, parity, AVT before pregnancy, HBeAg, AVT during pregnancy, HBVDNA viral load, cesarean section, treatment status postpartum.

**Table 4:** Subgroup analyses of the association of ALT levels at delivery with postpartum ALT flares

Characteristics	No. of patients	OR (95% CI)	P value	P for interaction
**Age (years)**				0.0011
< 28	1198	1.04 (1.02, 1.05)	<0.0001	
≥ 28	1445	1.01 (1.01, 1.02)	0.0004	
**Parity**				<0.0001
≥ 1	557	1.00 (1.00, 1.01)	0.3496	
0	2086	1.02 (1.02, 1.03)	<0.0001	
**HBeAg**				<0.0001
positivity	2518	1.02 (1.02, 1.03)	<0.0001	
negativity	43	0.99 (0.95, 1.03)	0.6197	
**HBV DNA (Log_10_ IU/mL)**				0.3455
≤ 5	1664	1.02 (1.01, 1.03)	<0.0001	
> 5	979	1.02 (1.01, 1.02)	<0.0001	
**AVT before pregnancy**				0.4109
Yes	81	1.03 (1.00, 1.07)	0.0847	
No	2562	1.02 (1.01, 1.02)	<0.0001	
**AVT during pregnancy**				0.1342
Yes	1663	1.02 (1.01, 1.03)	<0.0001	
No	980	1.01 (1.01, 1.02)	0.0011	
**Cesarean section**				0.0031
Yes	1129	1.01 (1.00, 1.02)	0.0046	
No	1514	1.03 (1.02, 1.04)	<0.0001	
**Treatment status postpartum**				0.0093
Maintenance of AVT	264	1.00 (0.98, 1.02)	0.7597	
Cessation of AVT	1339	1.03 (1.02, 1.04)	<0.0001	
No AVT	980	1.01 (1.01, 1.02)	0.0011	

Note: Each stratification adjusted for all the factors (age, parity, AVT before pregnancy, HBeAg, AVT during pregnancy, HBVDNA viral load, cesarean section, treatment status postpartum) except the stratification factor itself.

## Abbreviations

HBV: Hepatitis B virus
HBeAg: Hepatitis B e antigen
ALT: Alanine aminotransferase
ULN: Upper limit of normal
AVT: Antiviral therapy
